# Mind-body control: a new perspective on motor neuron function

**DOI:** 10.1038/s41392-024-01922-0

**Published:** 2024-08-30

**Authors:** Maria-Luise Petrovic-Erfurth, Albena Jordanova

**Affiliations:** 1 Molecular Neurogenomics Group, VIB Center for Molecular Neurology, VIB, 2610 Antwerp, Belgium; 2 Molecular Neurogenomics Group, Department of Biomedical Sciences, University of Antwerp, 2610 Antwerp, Belgium; 3 Molecular Medicine Center, Department of Medical Chemistry and Biochemistry, Medical University-Sofia, 1431 Sofia, Bulgaria

**Keywords:** Neuroscience, Cellular neuroscience

A recent study published in *Nature*^1^ used an optogenetically-activated behavioral paradigm in fruit flies to demonstrate that individual motor neurons command their target muscles in a biased manner to execute body movements, based on the mechanosensory feedback preceding the movement. This finding contradicts the current postulate that activation of a specific motor neuron would always result in a pre-determined fixed muscle response, adding important new perspectives in the fields of neurobiology, biomechanics, and robotics.

When a dancing ballerina executes her turns, she typically controls her head position and fixes her gaze on a single orientation point. This spotting technique helps her to maintain balance and to prevent dizziness during her complex movements. But how is this mind-body control achieved from a neurobiological point of view?

All motor behavior requires signal transduction from the brain towards the appropriate target muscles. The efferent neurons conveying the signal from the central nervous system to the periphery are known as motor neurons (MNs). Upper MNs start in the cortex and brain stem from where they transmit neural signals via long axonal processes to interneurons and lower MNs in the spinal cord. Then, lower MNs carry the signal onto their respective target muscles, to which they connect via a terminal structure known as a neuromuscular junction (NMJ). Here, a neurotransmitter is released into the synaptic cleft, leading to the electrical activation and contraction of the muscle fibers. Lower MNs are therefore regarded as the final, common elements of the neuronal signal transduction chain necessary for the execution of movements.

Despite this classical hierarchical view on the composition of motor circuits, remarkably little is known about the exact contributions that individual neurons or muscles of the motor system make to achieve a specific movement. Dissection of such details is difficult because the nerves and muscles belonging to the sensorimotor system are anatomically intertwined, rendering them relatively inaccessible to isolated manipulation and observation. A recent study by Gorko et al.^1^ attempts to achieve exactly that: a better comprehension of the contribution of individual MNs to a complex movement by studying the neck MNs of the fruit fly *Drosophila melanogaster*. Those MNs continuously regulate the head position during flight, which is essential for optimal gaze and body stability during motion in the air, similar to the behavior of the dancing ballerina.

The fruit fly is a well-suited experimental model for motor control studies. Flies display several motor behaviors that can be studied in established setups.^2^ Favorable for electrophysiological and optogenetic studies is the fact that the fly ventral nerve cord (VNC)- the equivalent to the human spinal cord- is more accessible than the vertebrate counterpart due to the lack of vertebrae. Importantly, the anatomy of the *Drosophila* nervous system is well characterized, and many neurons can be labeled and manipulated on a single-cell level.^2^ Head movements of *Drosophila* are controlled by about 25 pairs of neck MNs innervating predominantly thoracic neck muscles^1^ that regulate head rotation around all three cardinal axes (roll, pitch, and yaw).

Gorko and colleagues utilized an elegant combination of optogenetics and behavioral analysis to monitor the head movements elicited by the isolated stimulation of individual neck MNs during flight. Flies tethered to a tungsten pin were allowed to move as if they were flying in an arena, monitored by two video cameras (Fig. 1a). During the flight, a small light-emitting diode (LED)-coupled optical fiber was placed directly over the fly’s neck in such a manner that the head could still move freely while most of the emitted red light could pass through the neck. Light flashes were then used to stimulate single neck MNs expressing the red-light responsive channelrhodopsin CsCrimson and the yellow fluorescent protein mVenus. The individual MNs were labeled stochastically by combining the split-Gal4 method^3,4^ with a heat-shock-driven flip-out technique. After completion of the behavioral test, the flies were dissected, immunohistochemically stained (mVenus), and imaged confocally to identify the MNs that had been activated during the respective experiments.

**Fig. 1:**
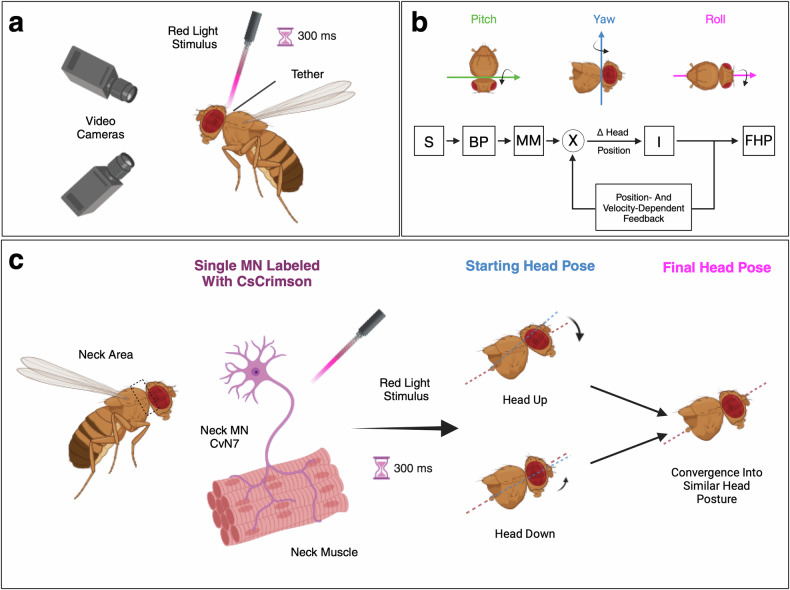
Activation of individual neck motor neurons (MNs) induces distinct posture-dependent movements that converge in a similar head position. **a** Experimental setup. Flies that express the red-light responsive protein Cs-Crimson in individual MNs are allowed to fly on a tether. Their head positions are monitored by two video cameras. An LED-coupled optical light fiber is placed closely over their neck and emits a red-light stimulus for 300 ms. **b** For each MN tested, the movement of the fly head around all three cardinal axes was recorded continuously. The observed head movements were fitted to a feedback model considering the motor neuron stimulus and the current pose of the head. The stimulus (S) is bandpass filtered (BP) and drives the neck muscle movement (MM). The resulting muscle drive is summed (⊗) with the position and velocity of the head, leading to an integrated (I) change (Δ) in head position determining the final head pose (FHP). **c** Activation of the same class of MNs leads to different muscle movements depending on the starting head pose. For example, stimulation of the MN CvN7 while the head pitched-up leads to a clear pitch-down movement. A movement in the opposite direction was however noted if the experiment started with the head in a pitched-down position. To better visualize the head pitch, orientation lines were drawn from the haltere to the antennae of the fly at the beginning of the experiment (blue) and at the end of the experiment (red). The figure was created with Biorender.com

The head movement of each fly was traced from the video recordings based on five fixed anatomical head landmarks^5^ and aligned in 3D using machine learning. The resulting measurements were visualized in “action field” plots, conveying the average three-dimensional rotational pose of the head at any given time scaled by magnitude. This special type of visualization became necessary because Gorko and colleagues found unexpectedly that stimulating a defined motor neuron does not lead to the execution of a stereotypical movement. Instead, movements of distinct directionality and speed were observed depending on the starting posture of the head. For example, when the MN CvN7 was activated while the head of the fly was pitched up, a clear pitch-down movement was recorded (Fig. 1c). If stimulation of the same neuron occurred when the fly head was pitched down, a weaker movement upward was noted. Importantly, the elicited motions were not random, and the neck movements always converged towards a neuron-specific target head pose. This phenomenon was apparent for all MNs tested, indicating that it might represent a general neck MN property allowing to cover the range of all possible head movements. These findings provide the basis for a novel inventory of the neck motor apparatus, mapping anatomy and connection between MNs and muscles, but also summarizing induced head rotations and speed of movements elicited.

As shown by mathematical modeling, the observed convergence of the MN-driven movements was more in line with a linear feedback model in which the individual MN action adds bias to a feedback loop responsible for centering the head (Fig. 1b). A feedforward model, representing the classical view that MNs elicit movements independent of starting head pose, did not perform well. Based on this, Gorko et al. explored the source of the postural feedback. In fact, they identified a single class of proprioceptive neurons, the lateral neck chordotonal neurons (LNCs), as important players of the feedback system in the neck control of *Drosophila melanogaster*.

Taken together, Gorko and colleagues’ data support the idea that MNs introduce a bias to existing continuous proprioceptive-motor loops. This is contrary to the common assumption that the brain can generate specific movements by simple activation of a fixed set of executing MNs, because the elicited motion changes with posture. It will be exciting to see whether vertebrate MNs also display a variable posture-dependent motion response and to dissect how individual MNs and their synaptic partners integrate information and contribute to a goal-directed movement.

## References

[1] Gorko, B. et al. Motor neurons generate pose-targeted movements via proprioceptive sculpting. *Nature* **628**, 596–603 (2024). 38509371 10.1038/s41586-024-07222-5

[2] Simpson, J. H. Descending control of motor sequences in Drosophila. *Curr. Opin. Neurobiol.* **84**, 102822 (2024). 38096757 10.1016/j.conb.2023.102822 PMC11215313

[3] Luan, H., Peabody, N. C., Vinson, C. R. & White, B. H. Refined spatial manipulation of neuronal function by combinatorial restriction of transgene expression. *Neuron* **52**, 425–436 (2006). 17088209 10.1016/j.neuron.2006.08.028 PMC1713190

[4] Pfeiffer, B. D. et al. Refinement of tools for targeted gene expression in drosophila. *Genetics* **186**, 735–755 (2010). 20697123 10.1534/genetics.110.119917 PMC2942869

[5] Lee, A., Kabra, M., Branson, K., Robie, A. A. & Roian, E. APT: Animal Part Tracker. *GitHub* https://github.com/kristinbranson/APT (2018).

